# Lipid II unlocked: strategies for obtaining a major antibiotic target

**DOI:** 10.1039/d5cc04843e

**Published:** 2025-10-17

**Authors:** Luke J. Tyrie, Milandip Karak, Stephen A. Cochrane

**Affiliations:** a School of Chemistry and Chemical Engineering, Queen's University Belfast David Keir Building, Stranmillis Road Belfast BT9 5AG UK m.karak@qub.ac.uk s.cochrane@qub.ac.uk

## Abstract

Antimicrobial resistance (AMR) is a major global concern. It caused nearly five million deaths in 2019 and is projected to be responsible for up to ten million annually by 2050. A deeper understanding of how antibiotics interact with their molecular targets is essential to addressing this threat, as it can facilitate rational drug design. One major antibiotic target is lipid II, a highly conserved and essential precursor in bacterial cell wall biosynthesis. As the final monomeric intermediate in peptidoglycan biosynthesis, lipid II has become an important target for antibiotic discovery. However, accessing lipid II remains technically challenging. In this review, we examine the three main strategies used to obtain lipid II: direct extraction from bacteria, enzymatic or chemoenzymatic assembly using purified or partially purified biosynthetic machinery, and total chemical synthesis. We discuss the strengths and limitations of each method, scalability, and structural control, and highlight notable approaches that are expanding the accessibility of lipid II and its analogues. These advances are critical not only for antibiotic research but also for understanding bacterial physiology at the molecular level.

## Abstract 2

Lipid II unlocked: a feature article comparing all practical strategies to access this essential antibiotic target.

## Graphical abstract

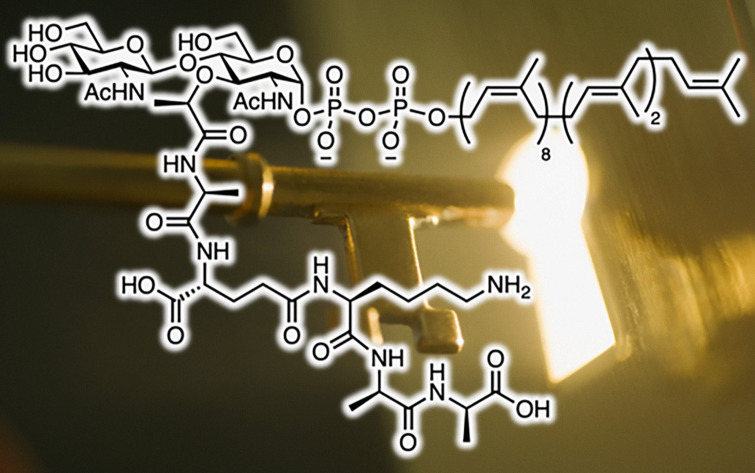

## Introduction

Antimicrobial resistance (AMR) continues to pose a serious threat to global public health.^1–3^ With nearly five million deaths associated with resistant infections in 2019 and projections suggesting up to ten million annually by 2050, AMR demands immediate and sustained attention.^4,5^ Resistance mechanisms, such as enzymatic degradation of antibiotics and mutations in target proteins, compromise the efficacy of existing treatments.^6,7^ As such, new antibiotics that are structurally and mechanistically distinct from existing classes are urgently needed.

**Fig. 1:**
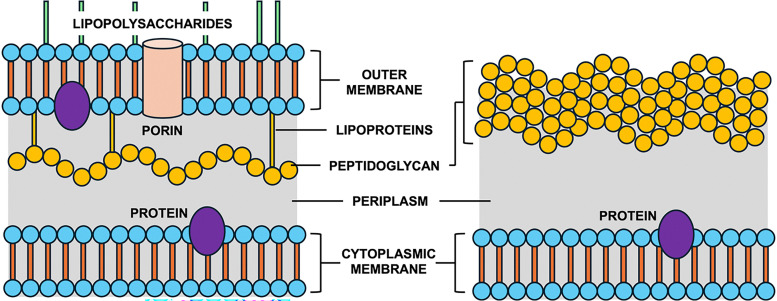
Cross sections of Gram-negative (left) and Gram-positive (right) cell walls. Gram-negative has an outer membrane with lipopolysaccharides and a thin peptidoglycan layer; Gram-positive has a thick peptidoglycan layer without an outer membrane.

Bacteria are composed of two major classes, Gram-positive and Gram-negative (Fig. 1). Whereas Gram-positive bacteria contain just a single cell membrane, Gram-negative bacteria have an extra layer of protection in the form of the outer membrane.^5,8^ This additional barrier blocks the entry of large scaffold antibiotics, making them much harder to treat. Antibiotics typically operate by one of five modes of action: inhibition of cell wall (peptidoglycan) biosynthesis, disruption of the cell membrane, inhibition of DNA and/or RNA synthesis, inhibition of protein synthesis at the ribosomes, and inhibition of folic acid biosynthesis (folate metabolism).^9^ Among these, inhibition of cell wall synthesis is the most common, demonstrated primarily by β-lactam and glycopeptide antibiotics.^10–12^ Penicillin, a β-lactam, covalently attaches to transpeptidase enzymes (penicillin-binding proteins), preventing peptidoglycan crosslinking and leading to bacterial cell lysis.^10^ Glycopeptides such as teicoplanin inhibit transglycosylation by binding the d-Ala-d-Ala terminus of peptidoglycan precursors.^13,14^

**Fig. 2:**
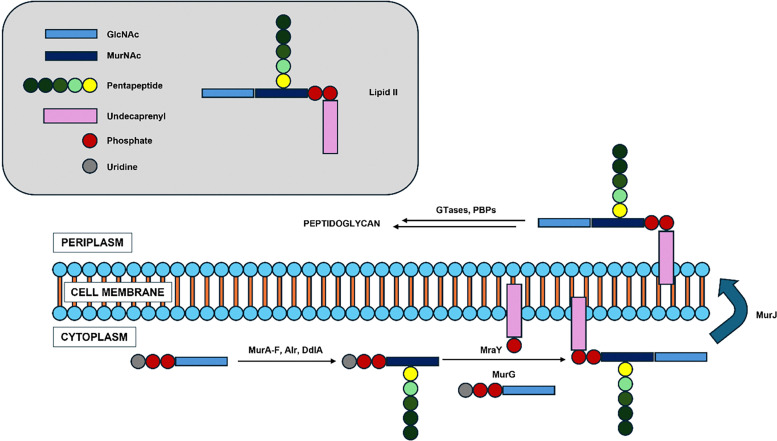
Schematic overview of the bacterial peptidoglycan biosynthesis pathway, highlighting the roles of peptidoglycan proteins (MurA-F, MurG, MurJ, MraY, and PBPs) at various stages.

Peptidoglycan, a key structural component of the bacterial cell wall, is a covalent, polymeric glycopeptide composed of alternating sugars interlinked by peptide chains.^15,16^ Its biosynthesis is highly conserved across bacterial species, making it an ideal target for antimicrobial agents (Fig. 2).^17–19^ The pathway begins with the conversion of UDP-GlcNAc to UDP-MurNAc *via* MurA and MurB.^20^ Subsequent ATP-dependent steps involving MurC-F ligate amino acids to form Park's nucleotide (UDP-MurNAc-pentapeptide).^20^ The d-Ala-d-Ala dipeptide is generated by Alr and DdlA from l-Ala.^21^ MraY then transfers the MurNAc-pentapeptide to undecaprenyl phosphate to form lipid I, followed by the addition of GlcNAc by MurG to yield lipid II.^20^ MurJ flips lipid II across the inner membrane,^22^ and GTases polymerize it into lipid IV,^23^ which is then crosslinked by PBPs and further polymerized to form mature peptidoglycan matrix.^20^

**Fig. 3:**
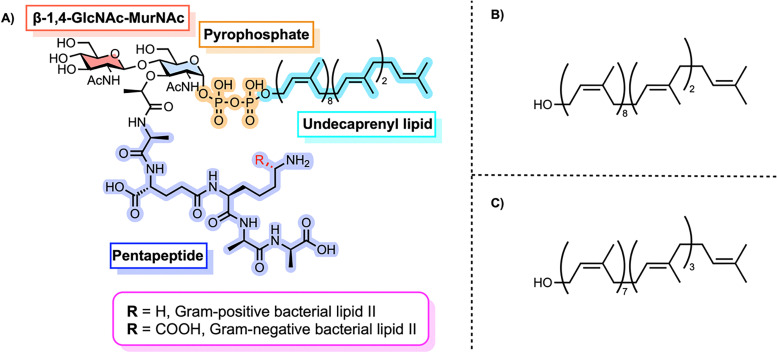
(A) Chemical structure of Lipid II, a membrane-anchored peptidoglycan precursor consisting of a disaccharide-pentapeptide connected to a polyprenyl chain through a pyrophosphate linkage. (B) Structure of bacterial undecaprenol. (C) Structure of plant undecaprenol.

Lipid II is the final monomeric intermediate in this biosynthetic sequence (Fig. 3A).^18,24,25^ It is a universally conserved molecule composed of a β-1,4-linked GlcNAc-MurNAc disaccharide attached to an undecaprenyl pyrophosphate lipid anchor, with a pentapeptide side chain on MurNAc.^25^ Bacterial undecaprenol has a (*Z*_8_,*E*_2_,ω)-configuration (Fig. 3B). Plant undecaprenol, which to the best of our knowledge has been used in all reported chemical and enzymatic syntheses, has the (*Z*_7_,*E*_3_,ω)-configuration (Fig. 3C). Lipid II isolated directly from bacteria therefore has the (*Z*_8_,*E*_2_,ω)-configuration but this is frequently mixed up in the literature as almost all lipid II prepared by other methods uses plant undecaprenol. The primary structural difference between Gram-positive and Gram-negative lipid II lies in the third amino acid of the pentapeptide stem: lysine in the former and *meso*-diaminopimelic acid (*m*-DAP) in the latter.^18^ It is worth noting that some Gram-positive bacteria, such as *B. subtilis*, also contain *m*-DAP (amide) at this position.^26^ In this feature, we use the identity of this third amino acid as the basis for distinguishing between Gram-positive and Gram-negative lipid II variants. Other variants, including those containing l-ornithine, d-lysine, or l-2,4-diaminobutyric acid, have also been reported.^27^

Due to its ubiquity and essential role in peptidoglycan biosynthesis, lipid II is targeted by many structurally diverse antibiotics.^25^ While the mechanisms of action for these antibiotics are beyond the scope of this feature, we refer the reader to several excellent literature reviews on the topic.^25,28–33^ Obtaining pure lipid II is essential for structural and mechanistic studies of both enzymatic processes and antibiotic interactions. However, its amphiphilic nature, chemical instability of the pyrophosphate linkage, and the structural complexity of the glycopeptide core pose substantial synthetic and isolation challenges. Even so, a wide array of functional derivatives of peptidoglycan fragments, including lipid I and lipid II analogues, has been reported, and we refer readers to comprehensive reviews covering their synthesis.^34,35^

This feature article covers the three main strategies for accessing lipid II and its analogues: direct bacterial extraction, enzymatic and chemoenzymatic synthesis, and total chemical synthesis. We emphasize the synthetic design, practical challenges, and significant developments that are broadening lipid II accessibility for structural, mechanistic, and drug discovery research.

## Direct bacterial extraction

**Fig. 4:**
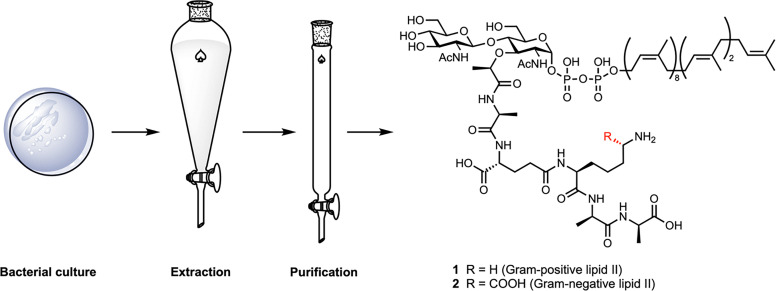
General workflow for extracting lipid II from bacterial cells.

Direct extraction of lipid II from bacterial membranes offers access to the native, unmodified molecule in its biologically relevant context (Fig. 4). However, this strategy is severely limited by the exceptionally low abundance of lipid II, estimated at only 1000 to 2000 molecules per cell,^36^ and its transient nature as an intermediate that is rapidly consumed during peptidoglycan biosynthesis. These challenges, combined with its amphiphilic character and tight membrane association, render direct isolation technically demanding and low-yielding. Nonetheless, direct extraction has been successfully applied in obtaining lipid II by several research groups.^26,36–38^

Pioneering work by Umbreit and Strominger in 1972 first demonstrated the feasibility of extracting lipid II directly from *E. coli*.^37^ Their protocol involved enzymatic digestion of frozen bacterial cells with lysozyme, followed by purification of lipid-linked intermediates using diethylaminoethyl (DEAE)-cellulose chromatography. The identity of the isolated lipid II was confirmed by mass spectrometry. A similar approach was employed two decades later by van Heijenoort *et al.*,^36^ who extracted both full-length and truncated (tripeptide-containing) forms of lipid II from *E. coli*. After initial purification on DEAE-cellulose, a second chromatographic step using silica gel improved the resolution of lipid II from other lipid intermediates.

More refined techniques have since been introduced to optimize extraction conditions and increase overall yields. In 2005, Guan *et al.* developed an isotope-labeling method using ^15^N-enriched media to generate ^15^N-labeled lipid II in *E. coli*.^38^ Exploiting the compound's solubility in a chloroform–phosphate-buffered saline (PBS) Bligh-Dyer system, they isolated lipid II directly from membranes and confirmed incorporation of eight ^15^N-atoms using electrospray ionization mass spectrometry (ESI-MS), consistent with the expected number of nitrogen atoms in the molecule.

**Fig. 5:**
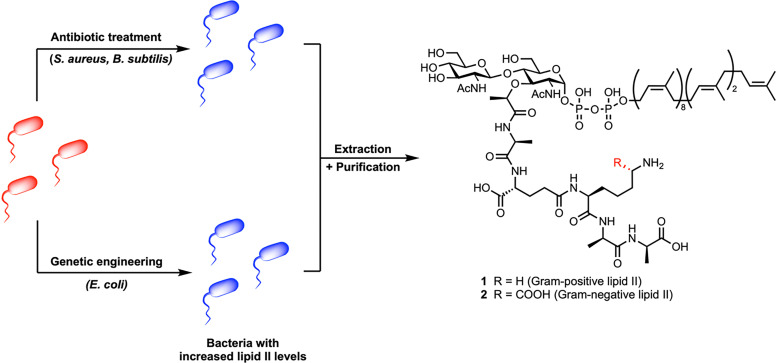
Strategies to increase intracellular lipid II levels and improve extractions.^26^

Building on the observation that lipid II accumulates when its downstream incorporation is inhibited,^39^ Kahne *et al.* devised a pharmacological strategy to increase its intracellular levels in *S. aureus*, *E. coli*, and *B. subtilis* (Fig. 5).^26^ Treatment of *S. aureus* with moenomycin, an inhibitor of peptidoglycan glycosyltransferases, led to a 10-fold increase in lipid II within 15 minutes, after which levels plateaued. In *B. subtilis*, which is intrinsically resistant to moenomycin, vancomycin was used instead, resulting in a 30-fold accumulation after 20 minutes;^40^ however, prolonged exposure caused cell lysis. Because the outer membrane of *E. coli* blocks access of both antibiotics to their targets,^41,42^ the authors engineered a conditional MurJ mutant in which the flippase could be inactivated by 2-sulfonatoethyl methanethiosulfonate (MTSES). This chemical inhibition arrested lipid II translocation, causing a 16-fold accumulation at the cytoplasmic leaflet of the inner membrane.

Despite these advances, direct extraction still yields sub-milligram quantities of lipid II from large culture volumes. The process encompassing membrane fractionation, organic extraction, and purification requires careful control to preserve the labile pyrophosphate moiety. While well-suited for structural or biochemical analysis of native lipid II, direct extraction remains impractical for analogue production or high-throughput applications. In such cases, chemoenzymatic or total synthesis provides more scalable alternatives.

## Chemoenzymatic and enzymatic approaches

**Scheme 1:**
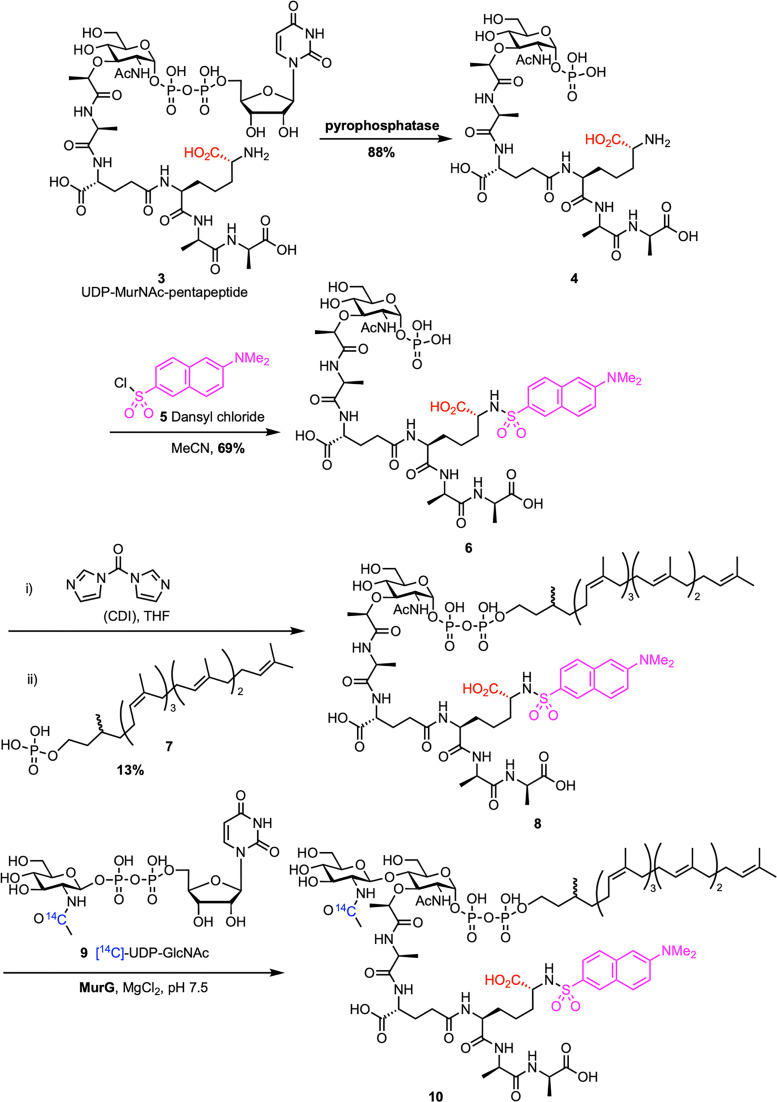
Chemoenzymatic synthesis of Gram-negative lipid II (10) by Blanot *et al.*^43^

Chemoenzymatic synthesis provides a reliable alternative to direct extraction of lipid II, enhancing scalability and enabling structural modifications. This approach typically involves chemically preparing lipid I analogues, which are then enzymatically converted to lipid II by the glycosyltransferase MurG. The first example of chemoenzymatic lipid II synthesis was reported by Blanot *et al.* in 1997 (Scheme 1).^43^ Here, the authors began by extracting UDP-MurNAc-pentapeptide 3 from *S. aureus*, following a procedure previously developed by Heijenoort *et al.*^44^ UDP-MurNAc-pentapeptide was converted to 1-phospho-MurNAc-pentapeptide 4 through treatment with pyrophosphatase. The *m*-DAP amino group on phosphate 4 was then dansylated with dansyl chloride 5, producing 1-phospho-MurNAc-pentapeptide(*N*-Dns) 6. Compound 6 was activated with CDI and coupled to dihydroheptaprenyl phosphate 7, yielding lipid I analogue 8. This lipid I derivative was subsequently transformed into lipid II analogue 10*via* the action of partially purified MurG and radiolabeled [^14^C]-UDP-GlcNAc 9. The formation of the product was confirmed by an increase in radioactivity in the lipid region during the MurG assay, indicating that lipid I analogue 8 served as a substrate for the enzyme. In a follow-up study, they optimized their assay conditions, which improved both the yield and purification of lipid II analogue 10.^45^

**Scheme 2:**
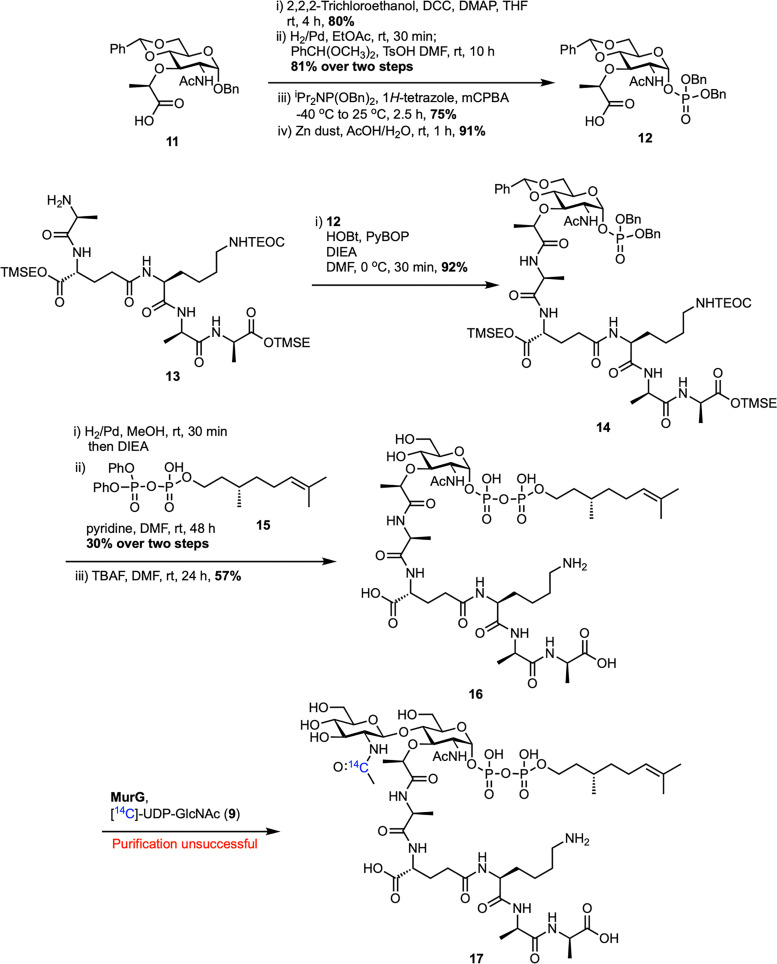
Attempted chemoenzymatic synthesis of lipid II (17) by Walker *et al.*^46^

**Scheme 3:**
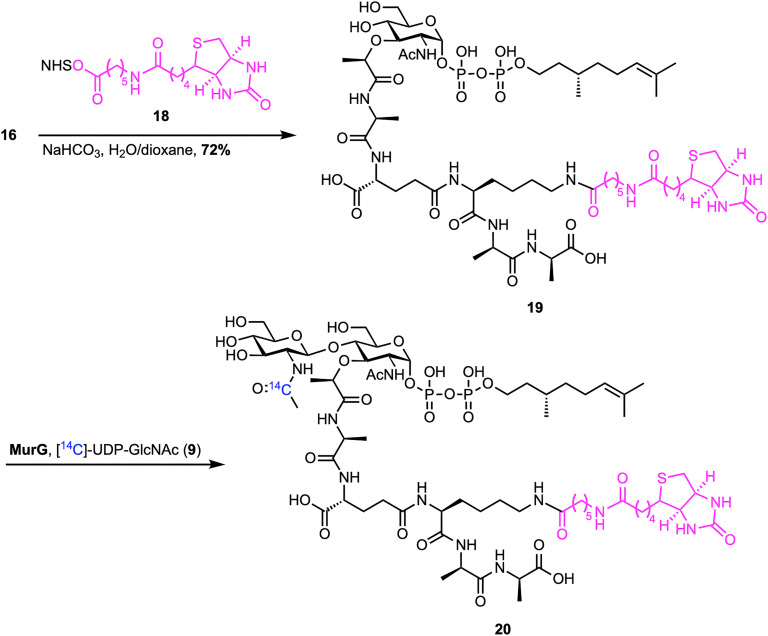
Biotinylation of lipid I and subsequent chemoenzymatic synthesis of biotin-labelled lipid II (20) by Walker *et al.*^46^

The following year, Walker *et al.* reported a reliable chemoenzymatic route to lipid II, starting from synthetic lipid I (Scheme 2).^46^ To prepare lipid I (16), commercially available MurNAc benzylidene 11 was converted into dibenzyl phosphate 12 through a four-step sequence. This process included protecting the carboxylic acid as a 2,2,2-trichloroethyl (TCE) ester, debenzylation of C1–OH, reprotection of C4–OH and C6–OH as a benzylidene, phosphorylation of C1–OH, and finally TCE removal. Dibenzyl phosphate 12 was then coupled with the silyl-protected pentapeptide 13, which was synthesized in 11 steps on d-Ala-Fmoc SASRIN^TM^ resin with an overall 15% yield, to afford benzylidene-MurNAc–pentapeptide dibenzylphosphate 14. Subsequent removal of the benzyl protecting groups, followed by coupling with (*R*)-(+)-*β*-citronellolOPO_3_PO(OPh)_2_15 and global deprotection, furnished lipid I analogue 16. Attempts to synthesize lipid II analogue 17 by treatment with MurG and [^14^C]UDP-GlcNAc proved challenging because the radiolabeled lipid II could not be separated from the [^14^C]UDP-GlcNAc starting material. To address this, the lysine amino group of lipid I analogue 16 was functionalized with biotin (18) and then converted into biotin-labelled lipid II 20 using MurG (Scheme 3). Despite the truncated lipid chain and biotin label, lipid I (19) was recognized as a substrate by MurG, but subsequent TGases did not accept the lipid II analogue 20.

**Scheme 4:**
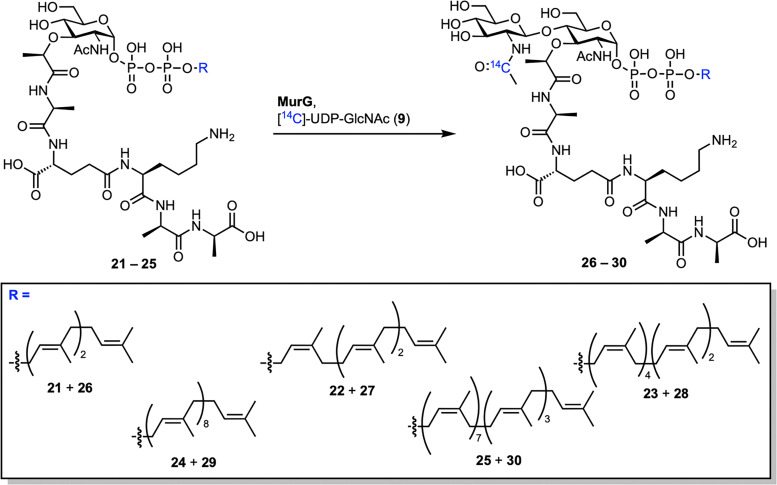
Extended substrate scope of MurG transformations by Walker *et al.*^47^

To improve substrate compatibility, the authors further optimized their chemoenzymatic route.^47^ They successfully synthesized a series of lipid I analogues (21–25) with different polyprenyl chains, including native Gram-positive lipid I (25) (Scheme 4). All lipid I derivatives were efficiently converted to their respective lipid II analogues (26–30) by MurG. Interestingly, native lipid I (25) reacted more slowly than other analogues, likely due to aggregation of the long undecaprenyl chain. Analogues with longer lipid chains served as substrates for TGase, with C_35_-lipid II (28) emerging as the best substrate under all tested conditions. Recently, Menche *et al.* reported a more scalable version of this chemoenzymatic synthesis, enabling multi-milligram production of farnesyl analogues of lipid I and II with improved reproducibility.^48^ Their modular approach integrates solid-phase peptide synthesis with efficient enzymatic glycosylation and was further extended to access a *S. aureus* lipid II analogue bearing the characteristic pentaglycine bridge.

Shortly afterward, Breukink *et al.* developed a simplified chemoenzymatic method for obtaining lipid II analogues with various polyprenyl tails to study nisin interactions.^49^ Instead of using purified MurG, they employed crude MraY and MurG from bacterial membrane vesicles to convert lipid I precursors into lipid II analogues, requiring only one chemical step—phosphorylation of polyprenols.^50,51^ The resulting polyprenyl phosphates were incubated with UDP-GlcNAc and UDP-MurNAc-pentapeptide in vesicles from *S. simulans* or *M. flavus*, producing native lipid II and analogues, which were purified through DEAE-cellulose chromatography. This modular approach generated a wide range of analogues, including short-chain (geranyl, farnesyl), medium to long-chain (11–25 isoprene units, mimicking bactoprenol), dolichol-type chains with saturated α-isoprene units, and even a non-prenylated C_20_-alkyl form, demonstrating MraY's notable substrate flexibility. Additionally, a pyrene-labeled fluorescent lipid II was also prepared, followed later by an NBD-labeled analogue.^52^ These probes revealed that lipid II does not flip spontaneously across membranes but requires a flippase enzyme.^22^ Overall, this chemoenzymatic method offered a scalable, high-yield route to obtain lipid II analogues, enabling direct studies of lipid II both as a nisin receptor and as an essential pore-forming component.

**Scheme 5:**
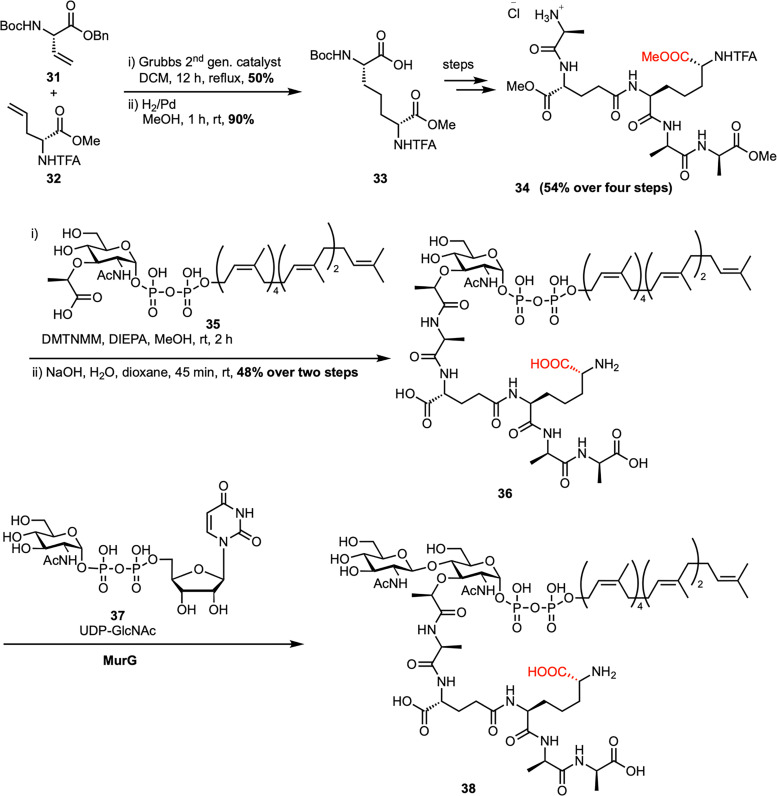
Chemoenzymatic synthesis of Gram-negative lipid II (38) by Kahne *et al.*^53^

Building on a modular chemoenzymatic approach, Kahne *et al.* developed a concise synthesis of Gram-negative lipid II containing *m*-DAP at the third position of the peptide stem (Scheme 5).^53^ The synthesis began with the chemical construction of orthogonally protected *m*-DAP (33) through olefin cross-metathesis of vinylglycine (31) and allylglycine (32), followed by hydrogenation. This key intermediate enabled the construction of the canonical *E. coli* pentapeptide (34), which was coupled to the lipid-linked MurNAc moiety (35, C_35_-PP-MurNAc) *via* DMTMM-mediated amide bond formation, followed by deprotection to afford lipid I (36) in good yield. Finally, enzymatic glycosylation with UDP-GlcNAc (37) catalyzed by MurG yielded the Gram-negative lipid II (38) in a single step. LC/MS analysis showed that *E. coli* PBPs can polymerize both Gram-positive (l-Lys) and Gram-negative (*m*-DAP) lipid II substrates, but only the Gram-negative version supports transpeptidase-mediated cross-linking due to its critical nucleophilic side chain at the third position.

**Scheme 6:**
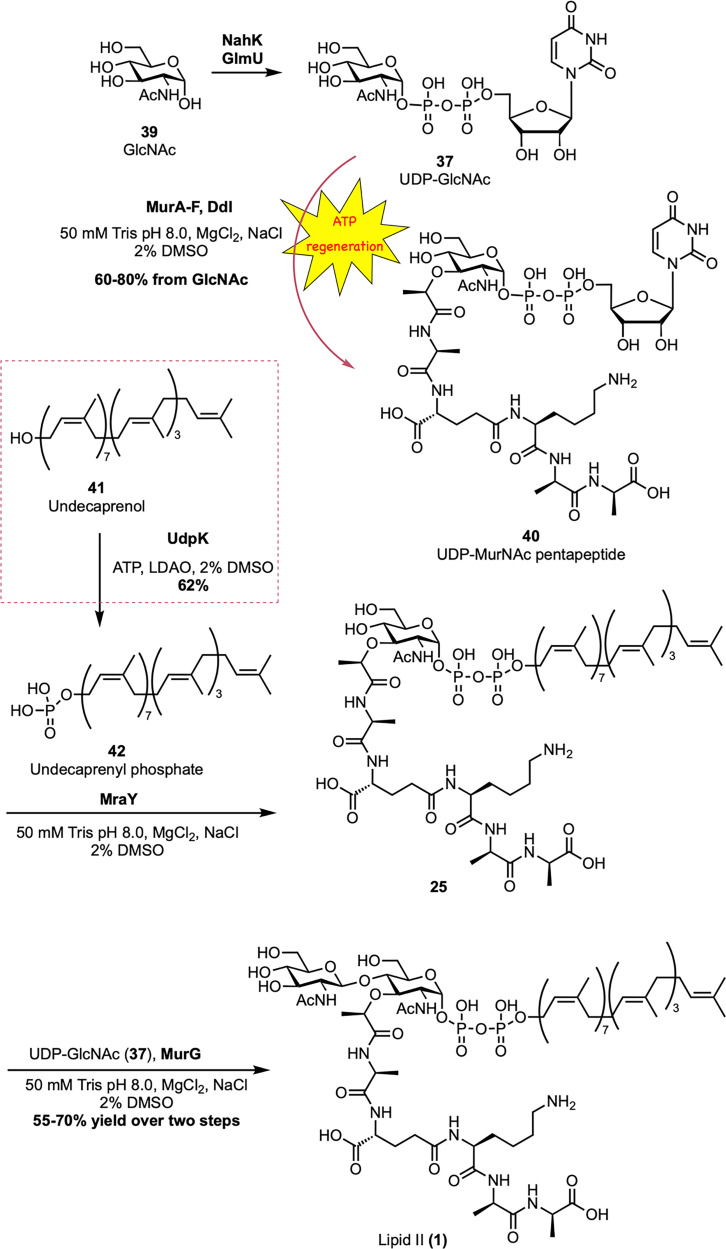
Enzymatic synthesis of Gram-positive lipid II (1) by Wong *et al.*^54^

The first total *in vitro* enzymatic synthesis of lipid II was reported by Wong *et al.* using purified recombinant enzymes and a cofactor regeneration system (Scheme 6).^54^ Undecaprenol (41) was enzymatically phosphorylated to undecaprenyl phosphate (42) by undecaprenol kinase (UdpK),^55^ an enzyme from *S. mutans*, with lauryldimethylamine oxide (LDAO) included to improve lipid solubility. The resulting lipid phosphate was coupled to UDP-MurNAc-pentapeptide (40) by MraY to produce lipid I (25), followed by MurG-catalyzed glycosylation with UDP-GlcNAc (37) to form lipid II (1). Both sugar nucleotides were produced enzymatically from GlcNAc (39) using NahK, GlmU, MurA–F, and Ddl, with ATP regeneration achieved by pyruvate kinase and phosphoenolpyruvate.^56–59^ The process, carried out either in sequential steps or as a one-pot reaction, achieved overall yields of 50–70%. Significantly, this modular platform allowed the synthesis of a diverse library of lipid II analogues by replacing undecaprenol with various isoprenoid alcohols.^54^ UdpK exhibited broad substrate specificity, accepting geraniol, farnesol, geranylgeraniol, phytol, dolichol, and others, which were subsequently incorporated into functional lipid II analogues *via* MraY and MurG. These analogues, especially those with geranylgeranyl and phytol tails, proved to be effective substrates for transglycosylases from *E. coli* and *C. difficile*, demonstrating the utility of this chemoenzymatic system for creating substrate probes.

Collectively, (chemo)enzymatic methods have established robust and scalable platforms for synthesizing lipid II and its analogues. These approaches have circumvented the inherent limitations of native lipid II isolation, such as low yield, heterogeneity, and structural instability, while enabling precise enzymatic transformations for substrate diversification, mechanistic understanding, and the development of high-throughput assays crucial to antibiotic discovery and peptidoglycan biosynthesis studies.

## Total chemical synthesis as a tool to access lipid II

In parallel with biosynthetically guided approaches, total chemical synthesis has emerged as a powerful and complementary method for producing lipid II with complete structural control. By assembling the molecule from manageable monosaccharide and peptide building blocks, this approach enables the incorporation of custom modifications at any position within the glycan, peptide stem, or lipid part. This level of control facilitates the development of non-natural analogues, isotopically labeled variants, and photoaffinity probes, which are essential for studying structure–activity relationships, investigating cell wall biosynthetic enzymes, and designing new antibacterial agents. Moreover, recent advances in protecting group strategies, chemoselective ligation techniques, and solid-phase synthesis have greatly enhanced the efficiency and scalability of lipid II total synthesis.

**Scheme 7:**
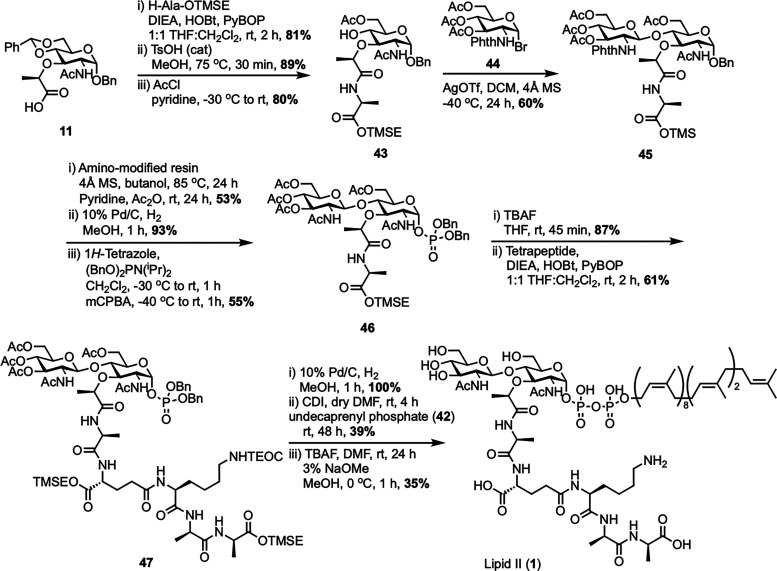
Total synthesis of Gram-positive lipid II (1) by Schwartz *et al.*^60^

Several different methods for the total chemical synthesis of lipid II have been reported so far. A notable synthesis was first described by Schwartz *et al.* at DuPont in 2001 (Scheme 7), marking a significant advance in constructing complex peptidoglycan intermediates.^60^ The synthesis began with MurNAc derivative 11, which was coupled to H-Ala-OTMSE to introduce the alanine residue to prevent unwanted cyclization at the lactyl position. After selectively opening the benzylidene protecting group at C4, secondary alcohol 43 was glycosylated with GlcNAc bromide donor 44 using AgOTf activation. A phthalimido group at the 2-position ensured the desired β-selectivity. This yielded phthaloyl-protected disaccharide 45, which was then deprotected and re-acetylated at the glucosamine residue. The anomeric position was unmasked and phosphorylated in a two-step, one-pot process, introducing a dibenzyl phosphate group (46). Following silyl deprotection, the free carboxylic acid was coupled to a protected tetrapeptide (γ-d-Glu(OTMSE)-l-Lys(TEOC)-d-Ala-d-AlaOTMSE), mimicking the natural peptide stem, using standard peptide coupling conditions to afford disaccharide pentapeptide dibenzyl phosphate 47. These bulky silyl groups helped control reactivity during the coupling steps but made final deprotection more challenging. Next, benzyl groups were removed by hydrogenation, and the resulting phosphate was activated with CDI and coupled to undecaprenyl phosphate (42) to install the lipid tail. Final global deprotection with fluoride and methanolysis removed remaining silyl and acetate groups, yielding lipid II (1) with an overall yield of 0.7%. Although the overall yield was low, this synthesis highlighted the importance of building lipid II using a modular approach by assembling the sugar, peptide, and lipid tail as individual components. More importantly, it enabled access to milligram-scale quantities of lipid II for the first time. This advance also made it possible to directly study the glycosyltransferase and transpeptidase activities of PBP1b, paving the way for deeper mechanistic insights and future antibiotic discovery.

**Scheme 8:**
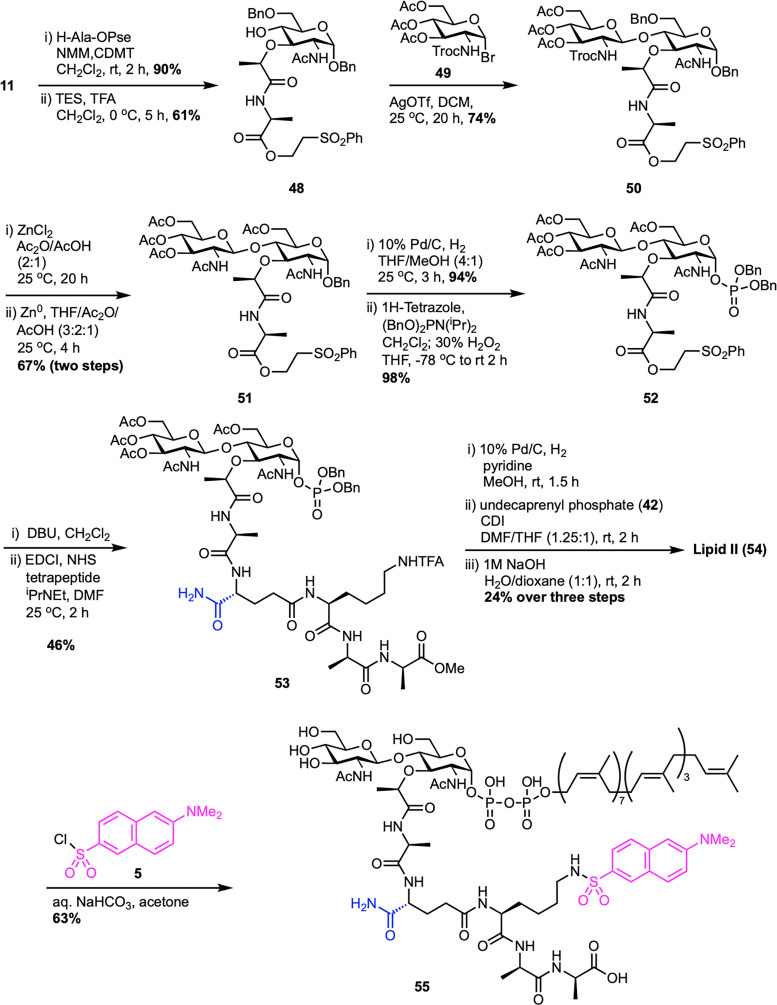
Total synthesis of Gram-positive lipid II (54) by VanNieuwenhze *et al.*^61^

Shortly after the groundwork established by DuPont, VanNieuwenhze *et al.* at Eli Lilly reported an improved route to Gram-positive lipid II (Scheme 8), achieving better efficiency and convergence.^61^ Their synthesis also began with the MurNAc intermediate 11, which was first esterified with H-Ala-OPse, followed by selective opening of the benzylidene protecting group at the C4 position to achieve the secondary alcohol 48. Silver triflate-promoted glycosylation between 48 and Troc-protected glucosamine bromide donor 49 furnished the disaccharide 50 with high β-selectivity.^62^ Strategic protecting group adjustments provided compound 51, followed by anomeric benzyl deprotection and phosphitylation, then oxidation to produce disaccharide dibenzyl phosphate 52. The Pse-ester was then removed under mild basic conditions to unmask the l-Ala carboxyl, which was converted into an active ester and coupled with a preassembled tetrapeptide using standard peptide coupling protocols to deliver 53, bearing acetate-protected hydroxyls, a methyl ester at the C-terminal d-Ala, and a TFA-protected Lys ε-amine, allowing for clean, orthogonal deprotection. This tetrapeptide, d-iso-Gln-l-Lys(ε-TFA)-d-Ala-d-Ala-OMe, was assembled in two steps by coupling Boc-d-iGln(NHS) with a protected l-Lys-d-Ala-d-Ala-OMe tripeptide,^63^ followed by acid-mediated Boc-deprotection. Next, benzyl phosphate 53 was deprotected by hydrogenolysis and activated as a phosphoroimidazolidate, enabling direct coupling with undecaprenyl monophosphate (42) under mild conditions. Final global deprotection using aqueous NaOH cleanly removed all base-labile groups to deliver lipid II (54) in 2% overall yield. Notably, this route improved the overall yield nearly threefold compared to the DuPont synthesis^60^ and eliminated the need for fluoride or hydrogenation-intensive steps for side-chain deprotection. The design of a triple orthogonal protection scheme, late-stage lipid installation, and a more streamlined tetrapeptide coupling process made this a scalable and synthetically elegant solution to lipid II access. Furthermore, the authors extended the utility of their synthetic lipid II by reacting 54 with dansyl chloride (5) to produce the fluorescent analogue Dns-lipid II 55, thus enabling applications in membrane-binding and enzymatic assays.

**Scheme 9:**
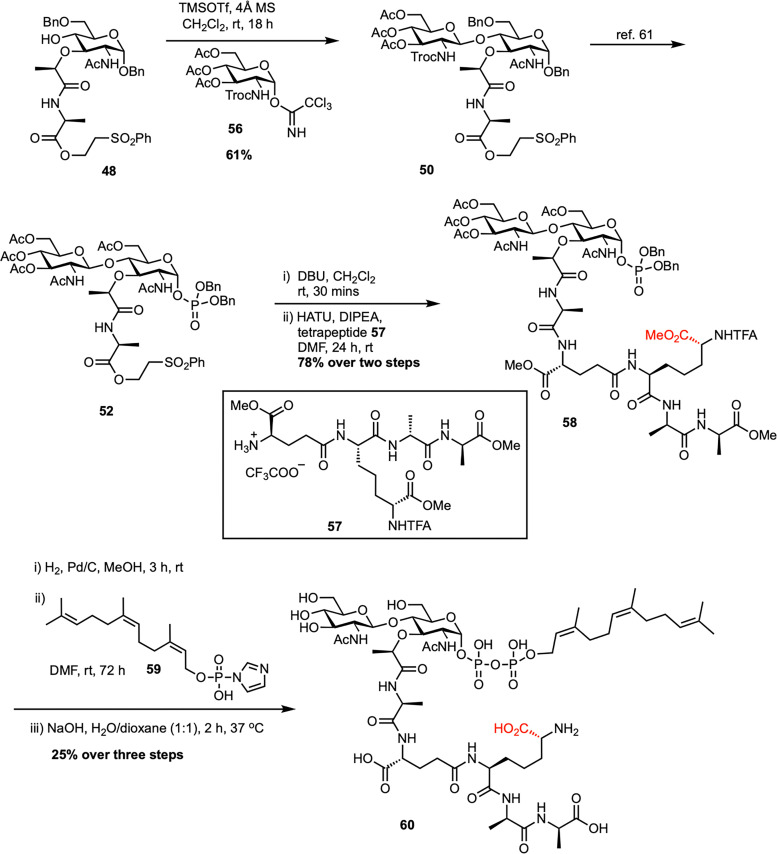
Synthesis of farnesyl-Gram-negative lipid II (60) by Vederas *et al.*^68^

The Eli Lilly synthesis of Gram-positive lipid II has proven to be a robust and adaptable platform, enabling the production of structurally diverse lipid II analogues through its modular design. Key to this approach is the use of disaccharide phosphate intermediate 52, which allows for flexible late-stage attachment of peptide chains and lipid tails. This flexibility has led to various applications that are adaptable across multiple contexts among various research groups.^64–67^ In 2016, Vederas *et al.* reported a modified synthetic route to access farnesyl Gram-negative lipid II (60), introducing changes to the polyprenyl tail by replacing the native undecaprenyl chain with a shorter, more tractable farnesyl group (Scheme 9).^68^ This work, as part of SACs doctoral studies in the Vederas lab, established a practical strategy for preparing lipid II analogues and enabled detailed studies of their interaction with the antimicrobial peptide tridecaptin A_1_. A key step in the synthesis was a TMSOTf-promoted glycosylation, coupling known acceptor 48 with Troc-protected trichloroacetimidate glucosamine donor 56 to afford disaccharide intermediate 50 in good yield. This intermediate was further transformed into disaccharide dibenzyl phosphate 52*via* the Eli Lilly synthetic route.^61^ The tetrapeptide 57 was synthesized following the protocol developed by Kahne *et al.*,^53^ and coupled with the deprotected l-Ala carboxyl intermediate synthesized from 52 to yield the corresponding disaccharide–pentapeptide phosphate 58. Final coupling with activated farnesyl phosphate 59, followed by global deprotection, delivered the target farnesyl Gram-negative lipid II (60) in 19% overall yield from 52. Later, during SACs postdoctoral work in the Davis group, a similar strategy was adopted to synthesize Gram-positive lipid II (1) for developing tunicamycin analogues and investigating nucleoside antibiotics scaffolds.^69^

**Scheme 10:**
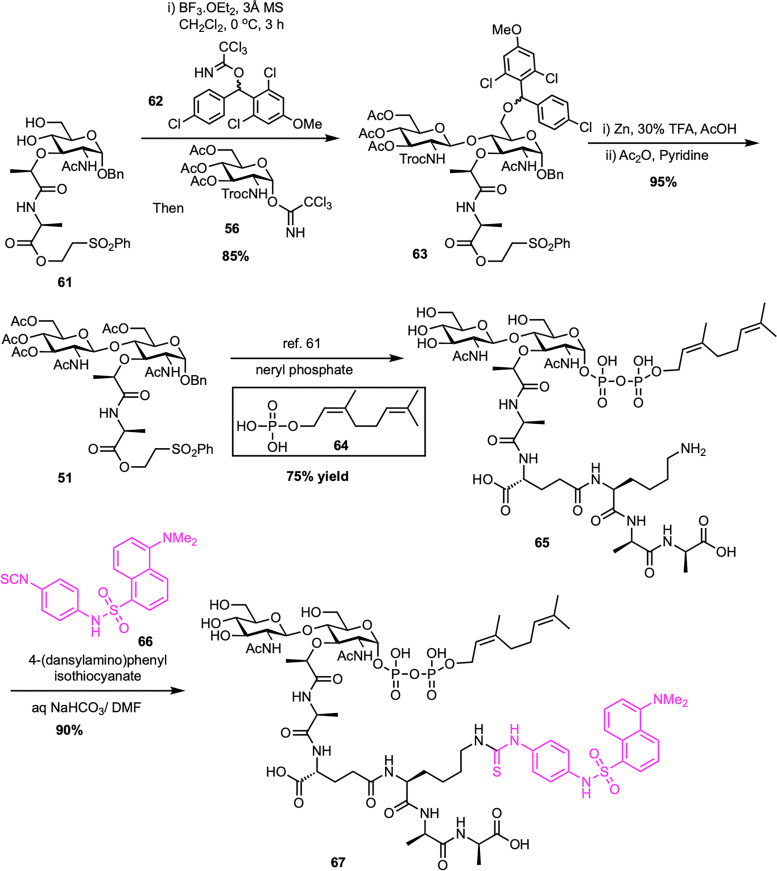
A new synthesis of neryl-lipid II (65) by Kurosu *et al.*^70^

In 2014, Kurosu *et al.* reported a noteworthy synthetic strategy that enabled the efficient preparation of lipid II and its neryl analogue (Scheme 10).^70^ Their method used a one-pot protection–glycosylation approach to simplify the synthesis of disaccharide intermediate 63. By employing the MDPM-imidate donor, (2,6-dichloro-4-methoxyphenyl)(2,4-dichlorophenyl)methyl trichloroacetimidate (62), as a selective C6-protecting group, they achieved high-yield glycosylation with GlcNAc trichloroacetimidate donor 56 under mild conditions, circumventing the limitations of earlier low-yield protocols.^60^ This strategy allowed the direct conversion of diol acceptor 61 into the disaccharide intermediate 63 with yields of up to 85%, using both solution-phase and solid-phase (resin-supported) methods. The *N*-Troc and C6-ether protecting groups were removed under acidic reductive conditions to give the corresponding amino alcohol, which was then acetylated to afford compound 51 in high yield. Following a route similar to the Eli Lilly synthesis,^61^ including benzyl deprotection, α-selective phosphorylation, and sequential coupling with a tetrapeptide and a lipid tail such as neryl phosphate (64), led to the successful preparation of lipid II and its neryl analogue (65) in high overall yields. Furthermore, neryl-lipid II was modified into a fluorescent probe, neryl-lipid II-*N*^ε^-dansylthiourea (67), *via* reaction with 4-(dansylamino)phenyl isothiocyanate (66), enabling its application in TGase studies.

**Scheme 11:**
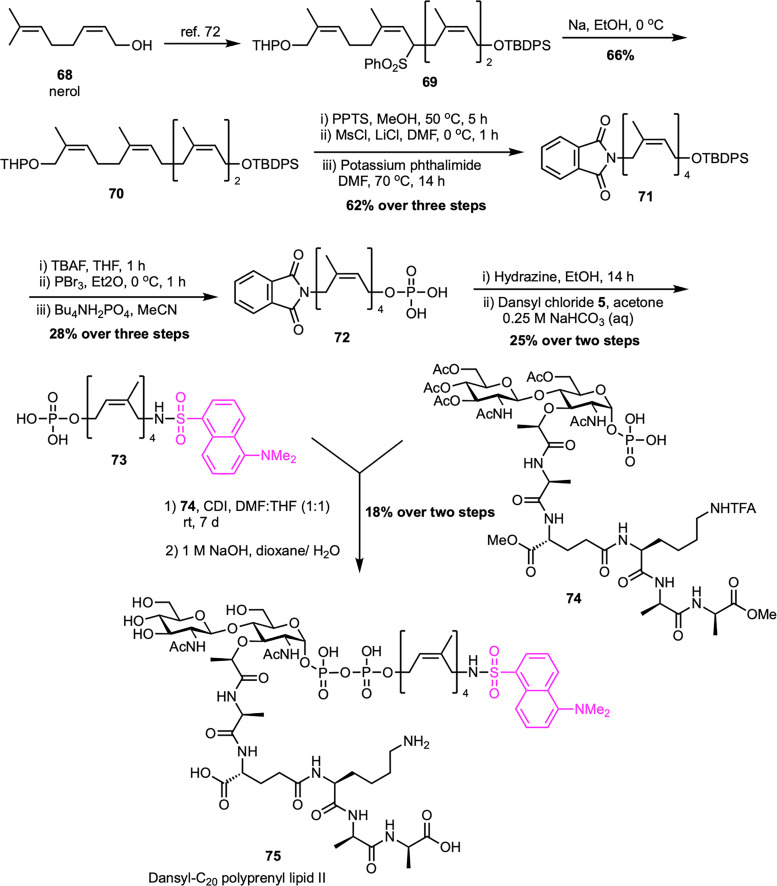
Synthesis of dansyl-C_20_-polyprenyl lipid II (75) by Cheng *et al.*^71^

Fluorescent probes for lipid II can be labeled not only at the peptide stem but also at the lipid tail, providing alternative strategies for enzymatic studies. In a notable study, Cheng *et al.* developed a concise and modular synthesis of a fluorescent lipid II analogue with a dansyl-labeled C_20_-polyprenyl chain, optimized for TGase assays (Scheme 11).^71^ The synthetic approach focused on a late-stage coupling between disaccharide–pentapeptide intermediate (74) and lipid phosphate (73) bearing a terminal dansyl group. The lipid portion was synthesized from known compound 69, obtained from nerol (68)^72^ through a sequence involving desulfonation to afford 70, followed by THP deprotection, phthalimide substitution, and phosphorylation to furnish 72 with moderate overall yields. Subsequent removal of the phthaloyl group and dansylation afforded the desired lipid phosphate 73. This was then coupled to the activated sugar–peptide unit 74, synthesized *via* the Eli Lilly route,^61^ and global deprotection provided the final fluorescent probe 75. Notably, placing the dansyl group on the lipid tail reduces interference with enzymatic recognition, allowing for direct HPLC detection without the need for radioactive labeling or enzymatic cleavage. The resulting probe proved to be an effective TGase substrate, facilitating the screening of inhibitors and the precise evaluation of their potency in biochemical assays.

**Scheme 12:**
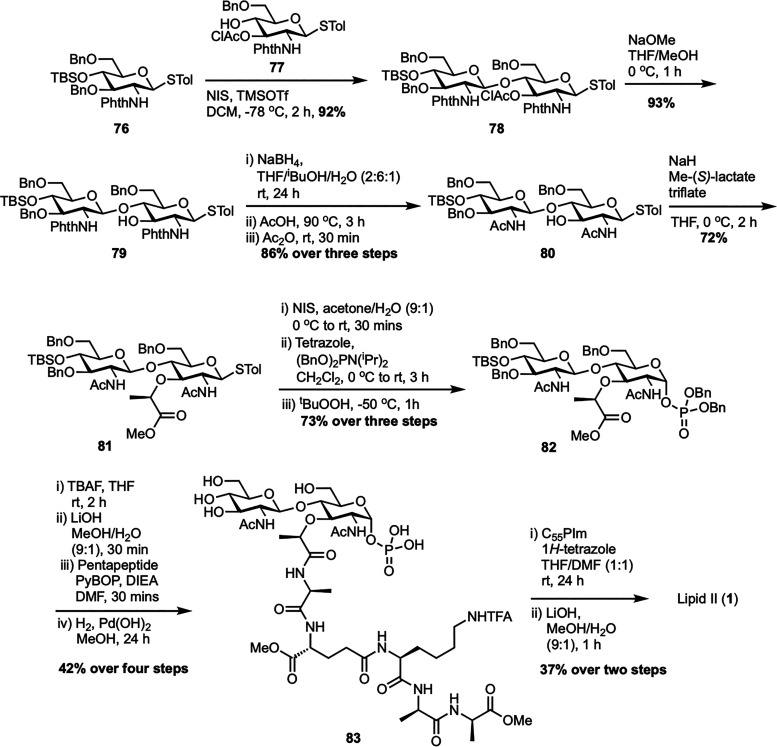
Total synthesis of Gram-positive lipid II (1) by Cheng *et al.*^73^

In 2011, Cheng *et al.* developed a new practical and convergent total synthesis of Gram-positive lipid II, providing an efficient route to bacterial TGase substrates (Scheme 12).^73^ Their strategy focused on the relative reactivity value (RRV) concept to build a key disaccharide intermediate,^74–76^ using glycosyl donor 76 (RRV = 134.1) and acceptor 77 (RRV = 9.2) to produce disaccharide 78 with high yield through optimized glycosylation. A major achievement was a three-step, one-pot process converting the *N*-phthaloyl group in 79 into an *N*-acetyl group, giving 80 in 86% yield. The synthesis continued with alkylation using methyl-(*S*)-lactate triflate^77^ to form corresponding ester 81, followed by thiocresol hydrolysis and anomeric phosphorylation to produce dibenzylphosphate 82. Following desilylation, hydrolysis of the methyl ester, coupling with the pentapeptide,^78^ and final debenzylation, the target disaccharide pentapeptide phosphate 83 was synthesized from compound 82 in four steps. Final coupling with activated undecaprenyl phosphoroimidazolidate (C_55_PIm) and global deprotection afforded lipid II (1) in 37% over two steps. The authors further expanded their study by synthesizing a series of lipid II analogues with modified peptide stems, ranging from truncated peptides to minimal, peptide-free, fluorescent, and lipid-tail variants, to investigate TGase activity.^79^ They discovered that while the terminal d-Ala-d-Ala was not essential for TGase interaction, the minimal active structure was d-Lac-l-Ala, with both methyl groups being critical.

**Scheme 13:**
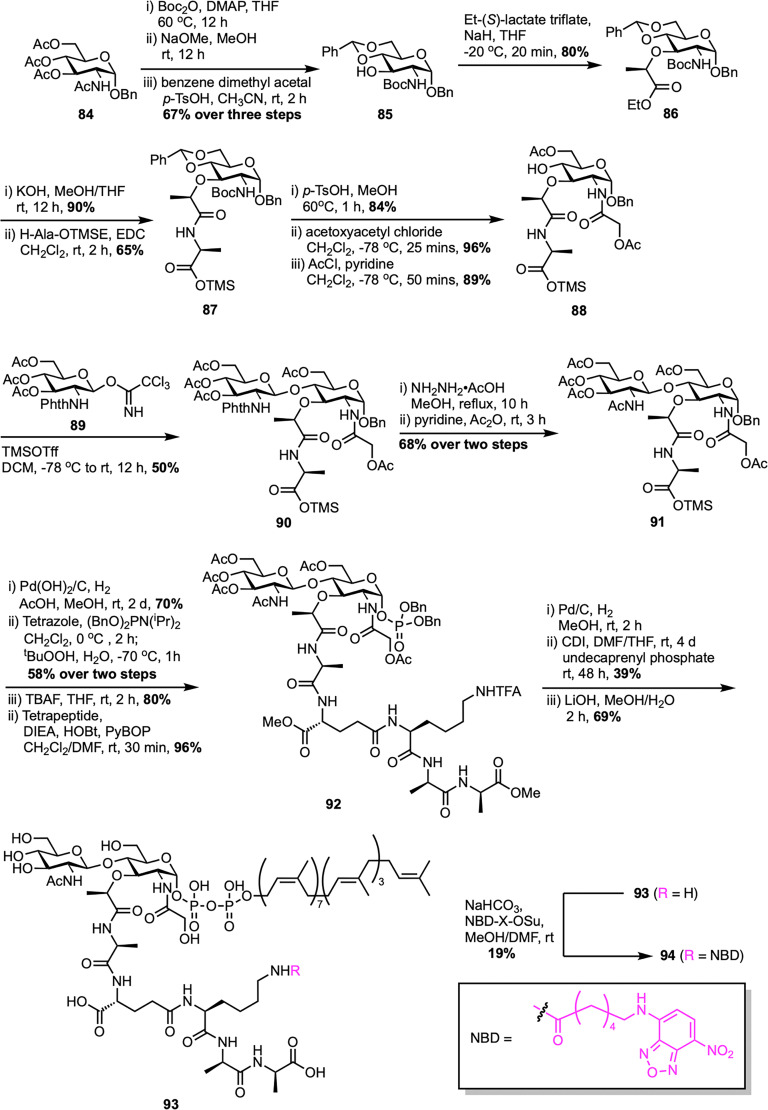
Synthesis of *N*-glycolyl-lipid II (93) by Cheng *et al.*^80^

In 2011, the same team described a method to synthesize an *N*-glycolyl analogue of *M. tuberculosis* lipid II, a vital substrate for mycobacterial transglycosylase in peptidoglycan biosynthesis (Scheme 13).^80^ The process began by converting d-glucosamine 84 into compound 85 through *N*-Boc protection, Zemplén deacetylation, and benzylidene acetal formation. Attempts to alkylate the C3–OH of compound 85 with *S*-(–)-2-chloropropionic acid were unsuccessful due to *N*-Boc removal, but *O*-alkylation with ethyl-(*S*)-lactate triflate succeeded, giving MurNAc ester 86 in high yield. Hydrolyzing ester 86, followed by coupling with the first amino acid of the pentapeptide chain using EDCI, yielded intermediate 87. Deprotecting the Boc-amine and benzylidene acetal with *p*-TsOH allowed the addition of the *N*-glycolyl group *via* acetoxyacetyl chloride, generating glycosyl acceptor 88. Glycosylation of acceptor 88 with donor 89 using TMSOTf formed disaccharide 90, which was then deprotected with hydrazine to remove the *N*-phthalimido group, and acetylated with acetic anhydride to yield disaccharide 91. Further transformations led to intermediate 92, which was converted into the lipid II analogue 93.^60,61^ In this modified Mtb *N*-glycolyl lipid II (93), the native decaprenyl phosphate and *m*-DAP groups were replaced with readily available undecaprenyl phosphate and d-lys, maintaining structural similarity and functional equivalence in the transglycosylase assay. The analogue was also tagged with a fluorescent probe (94) to evaluate its recognition by mycobacterial transglycosylase (MraY). This synthesis not only provided a functional analogue for studying mycobacterial cell wall synthesis but also supported the development of new antibiotics by demonstrating that simplified precursors can be used effectively.

**Scheme 14:**
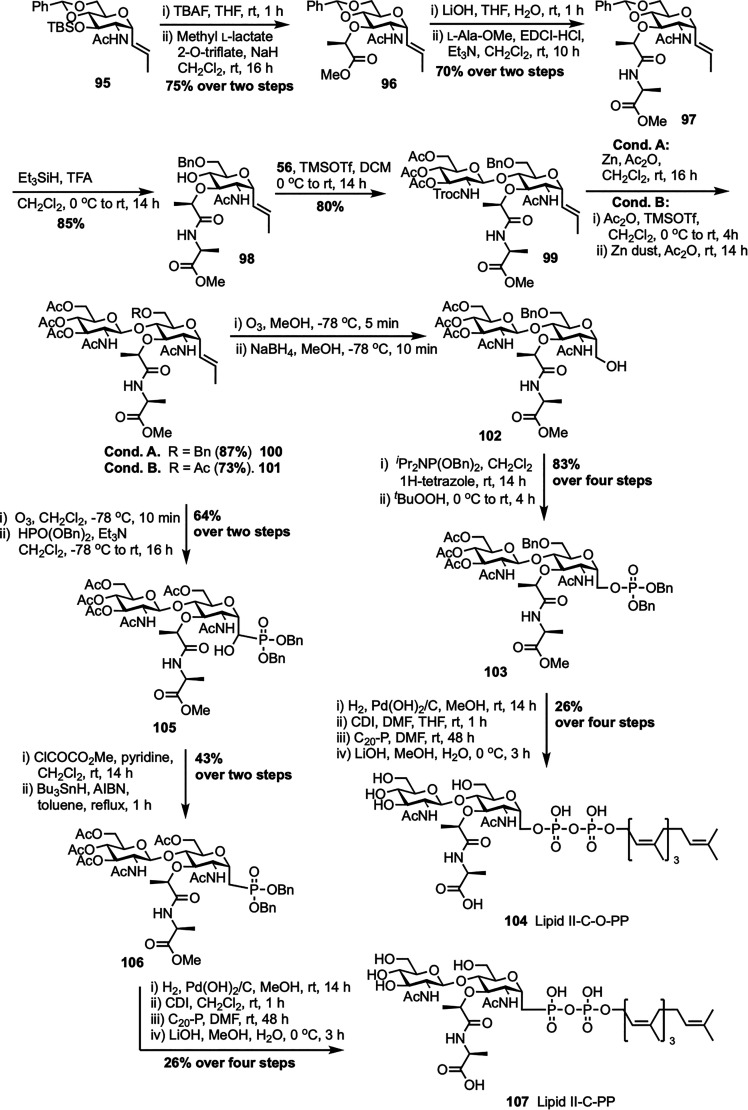
Synthesis of 1-C-glycoside-linked lipid II analogues (104 and 107) by Cheng *et al.*^81^

To design enzymatically stable lipid II analogues for bacterial TGase inhibition, Cheng *et al.* developed another new synthetic route to 1-C-glycoside-linked lipid II mimics containing either a sugar-phosphate or a sugar-phosphonate group (Scheme 14).^81^ The synthesis began with the isomerization of a 1-C-allyl precursor to 1-C-vinyl glycoside 95 using established methods,^82^ then proceeded with deprotection and *O*-alkylation to yield *N*-acetylmuramic acid derivative 96. This substrate was then converted to a peptide-bearing derivative 97, which underwent regioselective reductive ring-opening to afford glycosyl acceptor 98. Glycosylation of secondary alcohol acceptor 98 with Troc-protected glucosamine donor 56 yielded disaccharide 99, which was subsequently *N*-acetylated to form compound 100. For the synthesis of lipid II analogue 104, compound 100 was ozonolyzed and reduced to primary alcohol 102, phosphorylated to form dibenzyl phosphate 103, then debenzylated and activated with CDI. Coupling with tetraprenyl monophosphate (C_20_-P) and global deprotection resulted in lipid II-C-OPP (104) with a 26% yield from 103 over four steps. In contrast, the synthesis of the phosphonate analogue 107 required a modified route after the Arbuzov reaction failed with alcohol 102. Instead, vinyl glycoside 101 was ozonolyzed and reacted *in situ* with dibenzyl phosphite to give α-hydroxyphosphonate 105, which was deoxygenated *via* the Dolan–MacMillan approach to generate 1-C-phosphonate 106. Following debenzylation, CDI activation, coupling with C_20_-P, and global deprotection, lipid II-C-PP (107) was obtained with a 26% yield from 106 over four steps. Notably, while the final lipid II analogue with a 1-C-O-P linkage (104) showed significant TGase inhibitory activity (IC_50_ = 25 μM), its phosphonate counterpart (107) was inactive, illustrating how minor structural changes can greatly influence biological activity.

**Scheme 15:**
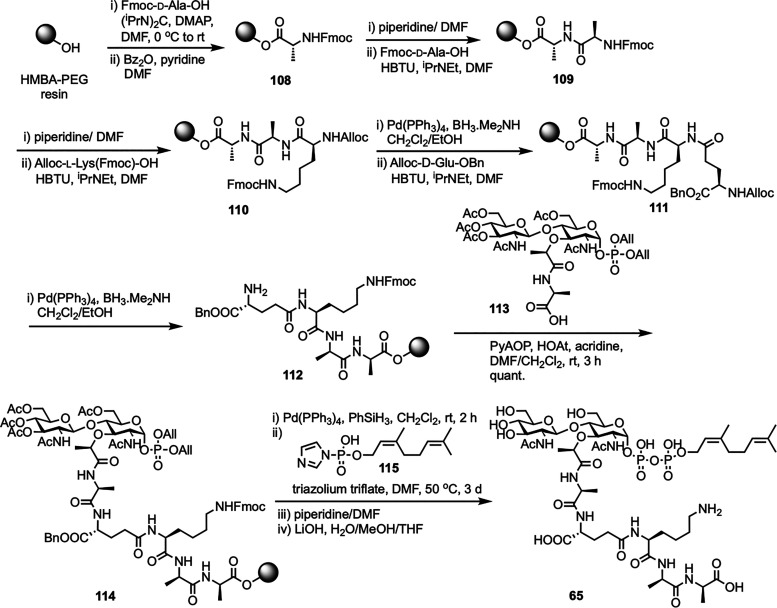
Solid-phase synthesis of neryl-lipid II (65) by Ichikawa *et al.*^83^

The first solid-phase synthesis of lipid II was reported by Ichikawa *et al.* in 2018, employing a modular 12-step strategy designed to efficiently construct structurally complex peptidoglycan precursors with minimal purification steps (Scheme 15).^83^ The synthesis started by loading Fmoc-d-Ala-OH onto HMBA-PEG resin to give 108, chosen for its broad solvent compatibility and hydrophilic properties. After benzoylation to cap unreacted hydroxyl groups, sequential Fmoc-based peptide couplings extended the chain to form dipeptide 109 and then a tripeptide 110 with Fmoc-d-Ala and Alloc-l-Lys(Fmoc)-OH, respectively. Allyloxycarbonyl (Alloc) deprotection followed by coupling with Alloc-d-Glu-OBn yielded the protected tetrapeptide 111, which was further deprotected to obtain the free amine 112. In parallel, the GlcNAc-MurNAc-Ala disaccharide phosphate 113 was synthesized in four steps from known disaccharide 51,^62^ involving catalytic hydrogenation to form a lactol, phosphorylation with diallyl phosphoramidite and 5-(benzylthio)-1*H*-tetrazole, oxidation with ^*t*^BuOOH, and DBU-mediated deprotection of the sulfonylethyl ester. This disaccharide phosphate was then coupled to the resin-bound free amine 112 to give disaccharide pentapeptide phosphate 114, a step that was complicated by potential epimerization at the MurNAc-Ala junction, which was successfully prevented using PyAOP/HOAt with acridine as a non-nucleophilic base. Clean Pd-catalyzed deallylation of 114 unmasked the phosphate, which was condensed with *in situ* generated neryl phosphoryl imidazolide 115 using a triazolium triflate activator at 50 °C, significantly enhancing diphosphorylation efficiency on solid support. Final Fmoc removal and aqueous base treatment completed the global deprotection and resin cleavage, affording neryl-lipid II (65) with an overall yield of 20%, demonstrating the effectiveness of solid-phase synthesis in assembling complex peptidoglycan frameworks. More recently, Menche *et al.* further advanced a synthetic strategy for farnesyl lipid II by incorporating non-canonical solid-phase peptide sequences that reflect the structural diversity among Gram-positive bacteria.^84^ Key improvements involved efficient sugar phosphate synthesis, a one-step process for preparing farnesyl phosphate, and a stereoselective solid-phase approach for assembling pentapeptides designed for species-specific variants.

**Scheme 16:**
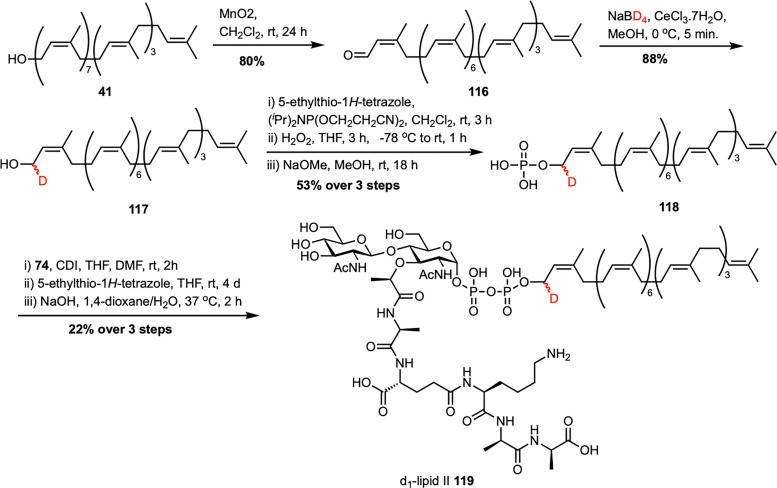
Synthesis of d_1_-lipid II (119) by Cochrane *et al.*^85^

In 2020, our team synthesized deuterium-labeled lipid II 119 (Scheme 16), creating a valuable probe to study bacterial cell wall biosynthesis and its antibiotic targets.^85^ Starting from undecaprenol extracted on a multigram scale from bay leaves, we identified the α-isoprene unit as the most practical site for introducing a stable isotopic label, chemically accessible yet still accepted by the enzymatic machinery that transforms undecaprenol into peptidoglycans. A streamlined sequence involving allylic oxidation of undecaprenol (41) gave the corresponding aldehyde 116, followed by Luche reduction with NaBD_4_/CeCl_3_, yielding d_1_-undecaprenol 117 in high yield. This intermediate was then converted into d_1_-undecaprenyl phosphate 118, which was coupled with an activated sugar-phosphate donor 74, prepared using our own protocol^69^ inspired by the Eli Lilly synthesis.^61^ After global deprotection, the final product, d_1_-lipid II 119, was obtained. A key advantage of this late-stage labeling strategy is its versatility, as a single isotopically labeled precursor can be directed into various undecaprenol-containing peptidoglycans, enabling kinetic isotope effect measurements, deuterium NMR studies, and detailed mechanistic insights into the enzymes responsible for bacterial cell wall biosynthesis. In our most recent study, we established a modular strategy for the total synthesis of lipid II and its short-chain analogues, with particular emphasis on optimizing the glycosylation step that forms the GlcNAc–MurNAc disaccharide core.^86^ Analogues prepared as part of this study were also used to investigate the mechanism of action of the novel lipid II-binding peptide Novltex.^87^

Finally, it is worth noting that Liu *et al.* reported an interesting synthetic route to a lipid II-like peptidoglycan oligomer (PGO) from chitosan, offering a major simplification over conventional multistep carbohydrate synthesis.^88^ Starting from low-molecular-weight chitosan, the C-2 amine was temporarily protected with a phthaloyl group, while triisopropylsilyl protection of the anomeric and primary hydroxyl groups improved solubility and synthetic control. Introduction of 2-bromopropanoate at C-3 generated alternating GlcNAc–MurNAc motifs, closely reproducing the natural repeating pattern of bacterial cell walls. Subsequent acetylation and amide coupling with a synthetic pentapeptide yielded a hybrid glycopeptide backbone, which was further elaborated through phosphorylation and lipidation to afford a tetradecanyl-linked pyrophosphate that closely mimics lipid II. The modular nature of this route also enables late-stage functionalization, exemplified by rhodamine conjugation *via* the lysine side chain of the pentapeptide, producing fluorescent PGOs that integrate efficiently into bacterial cell walls with minimal mammalian uptake, offering powerful tools for real-time imaging and bacterial diagnostics.

Together, these advancements showcase the flexibility of modern synthetic strategies in producing structurally diverse lipid II analogues. The resulting expansion of the chemical toolbox facilitates more precise investigations into bacterial cell wall biosynthesis and antibiotic mechanisms.

## Conclusion and future outlook

Accessing natural lipid II, along with unnatural and labelled variants, is essential in various fields such as antibiotic discovery and studying bacterial processes like growth, elongation, division, and sporulation. This feature outlines three main methods for obtaining lipid II: direct extraction from bacteria, enzymatic or chemoenzymatic assembly using purified or partially purified biosynthetic machinery, and total chemical synthesis. Each approach offers distinct benefits and challenges. The direct bacterial extraction, the earliest method developed, is arguably the most straightforward in terms of operation, mainly limited by the purification process. Its main drawbacks are that it yields only native lipid and the recovery from cultures is typically low (often less than a milligram). Both enzymatic and chemoenzymatic methods successfully produce milligram-scale amounts of natural, unnatural, and labelled analogues. Their versatility is supported by enzymes like UdpK, MraY, and MurG, which can tolerate varied lipid chain lengths and attach small chemical labels to lysine or *m*-DAP in the pentapeptide. However, these methods require high expertise to obtain the pure enzymes and are restricted by substrate scope. Additionally, organic synthesis skills are essential for the chemical steps involved. Total chemical synthesis offers exceptional control over the structural diversity of lipid II, limited mainly by available chemistries. Its modular design facilitates scalability; our team routinely synthesizes over 10 mg of lipid II analogues, with scale primarily limited by HPLC purification time. The main drawback is the high skill requirement in synthetic organic chemistry—even experienced chemists typically spend at least a month synthesizing lipid II from commercially available starting materials. Solid-phase synthesis could be the most efficient option, but it faces scale limitations due to resin-loading capacity and produces significant waste from reagent excesses. Ultimately, the method choice depends on the required quantity and type: small amounts of native lipid II are best obtained through bacterial extraction, unnatural and labelled variants *via* chemoenzymatic methods, and larger quantities of diverse analogues through total chemical synthesis. Looking ahead, the future of accessing lipid II depends on overcoming these practical challenges. Improving enzyme engineering, expanding substrate scopes, and incorporating automation into chemoenzymatic workflows could significantly improve efficiency and accessibility. Similarly, advances in solid-phase and greener synthetic methods will be vital for scaling up total synthesis while minimizing waste and complexity. A multidisciplinary approach combining synthetic chemistry, enzymology, and chemical biology will be crucial to unlocking new lipid II analogues and accelerating progress in understanding bacterial physiology and developing next-generation antibiotics.

## Conflicts of interest

There are no conflicts to declare.

## Data Availability

This is a review so data availability statement is not relevant.
